# Impact of Ionic Strength of Carrier Liquid on Recovery in Flow Field-Flow Fractionation

**DOI:** 10.1007/s10337-018-3551-z

**Published:** 2018-06-14

**Authors:** Tomasz Kowalkowski, Mateusz Sugajski, Bogusław Buszewski

**Affiliations:** 1 Department of Environmental Chemistry and Bioanalytics, Faculty of Chemistry, Nicolaus Copernicus University, Gagarina 7, 87-100 Torun, Poland; 2 Interdisciplinary Centre of Modern Technology, Nicolaus Copernicus University, Wileńska 4, 87-100 Torun, Poland

**Keywords:** Flow field-flow fractionation, Liquid carrier, Nanoparticle separation

## Abstract

Asymmetrical flow field-flow fractionation (AF4) and hollow-fiber flow field-flow fractionation (HF5) are techniques widely used in analytical, industrial and biological analyses. The main problem in all AF4 and HF5 analyses is sample loss due to analyte–membrane interactions. In this work the impact of liquid carrier composition on latex nanoparticles (NPs) separation in water and two different concentrations of NH_4_NO_3_ was studied. In AF4, a constant trend of decreasing the size of 60 and 121.9 nm particles induced by the ionic strength of the carrier liquid has been observed. In contrast, an agglomeration effect of the biggest 356 nm particles was observed when increasing ionic strength, which induced a significant drop of recovery to 35%. H5F provides better resolution and intensified peaks of NPs, but careful optimisation of system parameters is mandatory to obtain good separation.

## Introduction

Nowadays flow field-flow fractionation (F4 or FlFFF) is one of the most widely used analytical techniques for the separation and characterization of nano- and micrometer particles [1–4]. This technique uses a laminar stream of liquid flowing along the separation channel and a perpendicularly directed cross flow to separate the particles according to their size. Since the invention of FFF by Giddings [5], and later combination with DLS [6] this technique has been used for environmental, food and medical purposes [7–9].

**Fig. 1:**
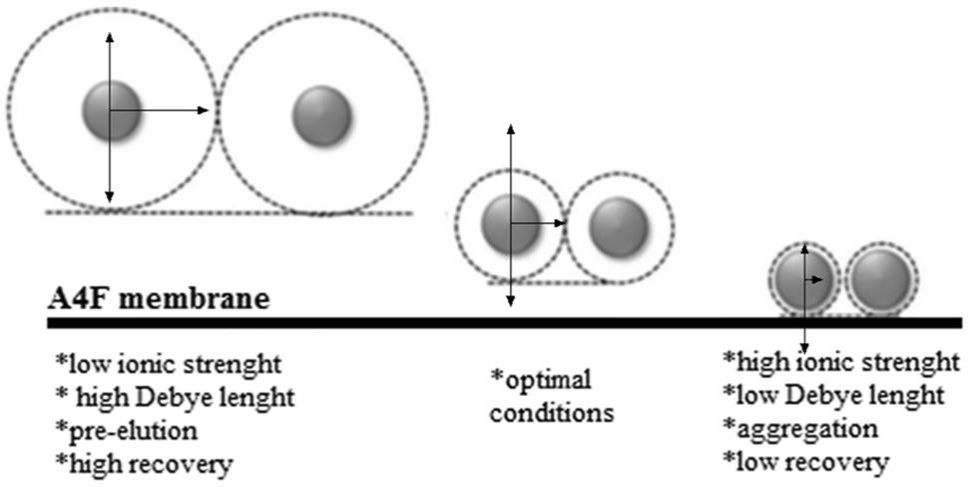
Changes of EDL depending on the ionic strength. Modified after [16]

Optimizing the separation of the 4F instrument in term of resolution requires setting up several parameters including: cross flow and channel outflow, relaxation and elution time as well as channel thickness and liquid carrier composition [10–12]. The last one is undoubtedly crucial for all FFF techniques. The optimum composition should stabilize the particles in unchanged forms, thus avoiding their agglomeration or sedimentation. Moreover, the ideal carrier liquid should not interfere with both the analytes and the membrane at the bottom of the channel. Three parameters can be changed: composition, ionic strength and pH. These ones have a direct effect on the electric double layer (EDL) (Fig. 1) of micro sized and nanoparticles [13–15].

EDL thickness can be characterized using Debye length. For aqueous solutions at 25 °C it is given by the formula:

1 $${K^{ - 1}}=0.304\frac{1}{{\sqrt I }},$$

where ${K^{ - 1}}$ is Debye’s length (nm) and *I* is the ionic strength of the solution expressed as:

2 $$I=1/2\mathop \sum \limits_{{i=1}}^{n} {c_i}z_{i}^{2},$$

where *c*_*i*_ is the concentration of each ion component (mmol dm^−3^), *z*_*i*_ is the ionic charge.

*K*^−1^ is dependent solely on the composition of carrier liquid, the properties of the membrane do not have effect on the value of *K*^−1^ [17].

If the sample contains charged particles then a mobile phase with higher ionic strength should be used [18]. It should compress EDL thickness. However the reduced EDL thickness allows the analyte to move closer to the membrane surface. This may potentially cause irreversible adsorption of the analyte on the membrane and, therefore, its loss during separation [18]. The ionic strength of mobile phase should be chosen to minimize possible losses and prevent premature elution.

The current study focuses on the influence of the ionic strength of the carrier liquid on recovery rate in two F4 sub-techniques: asymmetrical flow field-flow fractionation (AF4) and hollow-fiber flow field-flow fractionation (HF5).

## Elution Mechanism

The asymmetric flow field-flow fractionation theory is widely described in literature [19–21]. To fully understand the importance of ionic strength of carrier liquid, separation mechanism has to be discussed. Laminar flow of the liquid carrier with its parabolic velocity profile across the channel thickness transports particles to the channel outlet. After stressing the particles with external field (during relaxation step) distribution thickness of each component next to the accumulation wall is related to its diffusion coefficient. As a consequence of the two rival forces: transport by the transverse component of flow and diffusional flux, the steady-state cloud of particles is generated. If the transverse transport is not sufficient to counter the diffusional transport of the smaller particles then they will be distributed across the thickness of the channel. The laminar flow allows them to elute earlier, in contrast to those with a lower diffusion coefficient placed near the accumulation wall. This mode is called normal or Brownian elution mode [22, 23].

**Fig. 2:**
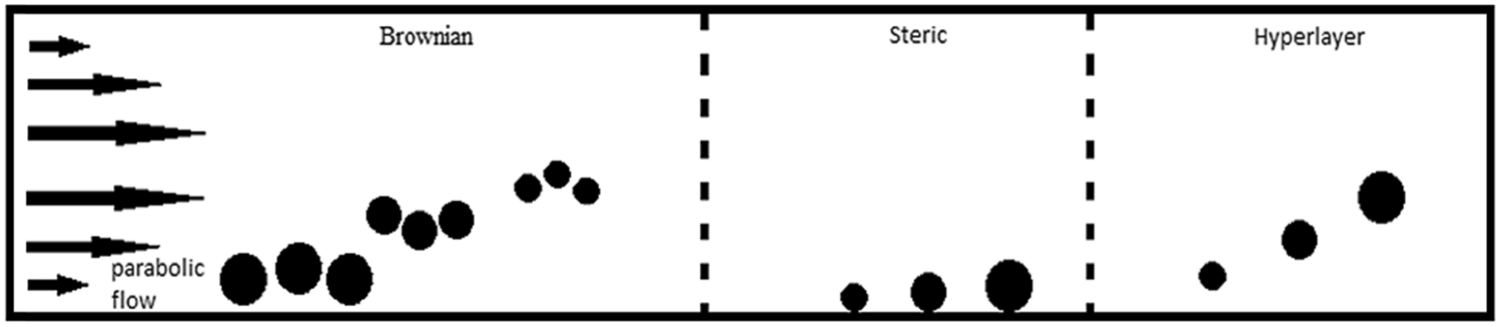
Elution mechanisms in FFF

For the larger particles the diffusion coefficient is very low, thus Brownian motions become irrelevant. Diffusion is not sufficient to counteract movement of particles generated by the cross flow. The thickness of the particles cloud is concentrated on the accumulation wall and is controlled by geometric dimension of the particles only. This mechanism of separation is called steric separation. Separation sequence is inverted in comparison to the normal mode. The particles are settling directly on the accumulation wall. Separation is affected by a hydrodynamic force, which disturbs their contact with the wall. Hydrodynamic force increases with higher flowrates. The direction of the hydrodynamic force is directed from accumulation wall. As a consequence the balance between the hydrodynamic lifting forces and a cross flow, the elution time of particles varies with different sizes. This mechanism is called hyperlayer separation. The order of elution is the same as for the steric separation. Those three elution mechanisms are depicted in Fig. 2 [24].

In reality however it is practically impossible to distinguish between the mechanisms of steric separation and hyperlayer separation. Both are closely related and give similar results. Moreover one can not predict the crucial condition of the inversion between normal and steric/hyperlayer mode. Usually, for polydispersed samples all of these mechanisms can occur simultaneously [24, 25].

## Materials and Methods

The water used in the study was prepared in the Milli-Q system (Millipore, USA). The ammonium nitrate pure for analysis was used for the preparation of the carrier liquid (Avantor Performance Materials, Poland). The study was conducted on Nanobead NIST traceable standard of polystyrene nanoparticles (NPs) purchased from Polysciences Inc. (Germany). The size of NPs were 60 (± 0.8), 121.9 (± 1.5) and 356 (± 4.5) nm.

Three carrier liquids were used in this study: Milli-Q water, 1 mM NH_4_NO_3_ and 10 mM NH_4_NO_3_. Every solution was prepared on the day of analysis. Before the analysis carrier liquid was filtered using 0.1 µm nylon membrane (Merck Millipore, Poland). To prepare second and third carrier 80.04 and 800.4 mg of NH_4_NO_3_ were diluted in 1 dm^3^ of Milli-Q. Concentration of latex nanobeads was as follows: 4 mg mL^−1^ for 60 and 121.9 nm and 0.8 mg mL^−1^ for 356 nm.

Measurement of the hydrodynamic diameter was conducted with Zetasizer Nano ZS (Malvern, UK) in flow mode using QS flow cell (Hellma Analytics, Germany). Particles of specified sizes were suspended in carrier liquids prior to analysis. Measurements were carried out at 20 °C within 30 cycles.

During the study the influence of the carrier liquid on the recovery was verified. For this purpose, two analyzes were performed for particles of known size suspended in one of the three carrier liquids. The first analysis did not include cross flow (so called direct elution to detectors). The second analysis was performed with cross flow. In both runs the detector flow rate was the same. The ratio of the area of peaks obtained on fractograms is considered to be recoverable.

3 $$R=\frac{A}{{{A^*}}} \times 100\%$$

where *A* is the peak area of the nanoparticles with added cross flow, A* is the peak area without cross flow (reference run).

## AF4 and HF5 Conditions

AF2000 Multi Flow system (Postnova, Germany) can work in two modes AF4 and HF5. The operating system depends on the channel used. In AF4 mode, the system was equipped analytical channel having 335 and 60 mm, length and width respectively and 350 µm spacer. 10 kDa regenerated cellulose was used as a channel membrane. For HF5 commercial hollow fibre cartridge (HF-28AN by PostNova) was used. The tube diameter was 800 µm and membrane cut-off was 10 kDa. Three detectors: MALS (multi-angle light scattering), DLS (dynamic light scattering) and UV–Vis were used for signal acquisition. Before analysis the sample was mixed using ultrasonic bath and vortex. The UV signal of the analyte was recorded at a wavelength of 280 nm. All the analyses were performed after setting the appropriate flow rates and stabilization of pressure in the channel. Analyses were repeated three times and averaged fractograms were shown in “Results and Discussion”. Evaluation of the MALS signal was performed using AF2000 Control software. As a computational method, a model for spherical particles was used. Molecular mass of each peak in all samples were determined. The membrane and cartridge were exchanged on average every 30 analyzes or when the signal was unstable or weak. The parameters used in the analysis are listed in Table 1.

**Table 1:** AF4 and HF5 analysis parameters

Step	Purpose	Duration (min)	AF4	HF5
FOCUS	Stabilisation of channel and detectors. Injecting and focusing latex nanobeads. Formation of latex nanobeads lines	5	Delay time: 1 min	Delay time: 1 min
			Injection flow: 0.20 mL min^−1^	Injection flow: 0.20 mL min^−1^
			Injection time: 4 min	Injection time: 4 min
			Cross flow: 1.2 mL min^−1^	Cross flow: 0.75 mL min^−1^
			Focus pump: 1.5 mL min^−1^	Focus pump: 0.93 mL min^−1^
ELUTION	Separation	110	Cross flow: 1.2 ≥ 0 mL min^−1^	Cross flow: 0.75 ≥ 0 mL min^−1^
			Focus pump: 1.5 ≥ 0 mL min^−1^	Focus pump: 0.93 ≥ 0 mL min^−1^
			Tip flow: 0.2 ≥ 0.5 mL min^−1^	Tip flow: 0.2 ≥ 0.5 mL min^−1^
RINSE	Washout of particles from channel and inject port	10	Tip flow: 0.5 mL min^−1^	Tip flow: 0.38 mL min^−1^

Comparing the parameters granted in Table 1 one can see that parameters for HF5 are a bit different due to usage of cartridge causing too high and unstable pressure in whole system when AF4 parameters are applied. The manufacturer suggests reducing the cross flow and inlet flow by 50 and 75% respectively, so the situation with unstable pressure can be avoided.

## Results and Discussion

### DLS Analysis

Nanoparticles of given diameters were suspended in three solutions: Milli-Q water, 1 and 10 mM NH_4_NO_3_. Nine samples (3 independent replicates) were analysed using DLS (Zetasizer Nano ZS, Malvern). Application of DLS allowed to measure hydrodynamic diameter. The results of the measurements are summarized in Table 2.

**Table 2:** Hydrodynamic size of analysed nanoparticles

Nominal size (nm)	Water (nm)	1 mM (nm)	10 mM (nm)
60	66.6 ± 0.7	63.6 ± 3.1	63.1 ± 0.7
121.9	145.8 ± 0.9	129.7 ± 0.6	121.9 ± 2.1
356	431 ± 8.1	533.5 ± 7.4	694.4 ± 17.9

Weak but the constant trend of decreasing the size of 60 nm particles induced by ionic strength of the carrier liquid has been observed. Similar trend of particles with a declared diameter of 121.9 nm was measured. Both nanobeads showed the largest EDL in the water. The biggest decrease occurs after changing to 1 mM NH_4_NO_3_. In this case, 10 mM is already apparent (1.46 ± 0.21% decrease).

In the case of the largest particles (356 nm) the change of mean particle size looks quite different. With the increase of ionic strength, EDL should be reduced, but we are observing a significant increase. Agglomeration effect or micellar layer growth was observed in all solutions. Higher ionic strength is enhancing these phenomena. The particles suspended in water have a diameter of about 431 nm. By increasing the ionic strength their diameter was approximately 694.4 nm, which was almost double the value declared. Similar effect was observed by Lang et al. [26]. They investigated influence of increased NaCl concentrations on retention shift of 25 and 50 nm polystyrene NPs in AF4.

The results obtained are consistent with the generally accepted theory of hydrodynamic diameter change due to ionic strength change of the carrier phase [27]. In case of changing the solution from water to 1 mM NH_4_NO_3_, the greatest difference in EDL change was observed. The difference between 1 and 10 mM is neglectable.

### Recovery Rate

Calculated recovery rates are presented in Table 3. Particles with size of 60 nm received the highest recovery in 10 mM NH_4_NO_3_ (85.56%). The lowest recovery was for 1 mM NH_4_NO_3_ (78.39%), 2% lower then the recovery in water. In this case particle-membrane interactions become more repulsive.

**Table 3:** Recovery rates of particles for three carrier liquids

Nominal size (nm)	Water (%)	1 mM (%)	10 mM (%)
60	80.5 ± 0.2	78.4 ± 0.31	85.6 ± 0.2
121.9	82.8 ± 0.2	73.1 ± 0.3	nd
356	87.8 ± 0.3	90.6 ± 0.51	35.2 ± 2.1

For 121.9 nm particles the situation was different. In this case, the recovery was calculated for water and 1 mM NH_4_NO_3_ only. By using 10 mM NH_4_NO_3_ as the carrier liquid, the interaction between the particles and the membrane was too high to acquire any detector signal. The highest recovery was achieved for water (82.83%) and was by 10% higher compared to the value measured in 1 mM NH_4_NO_3_ (73.05%).

Almost all recovery values are higher than 70%. Gigault et al. [16] reported that recovery higher than 70% can give a chance to make correct analysis with reliable results.

**Fig. 3:**
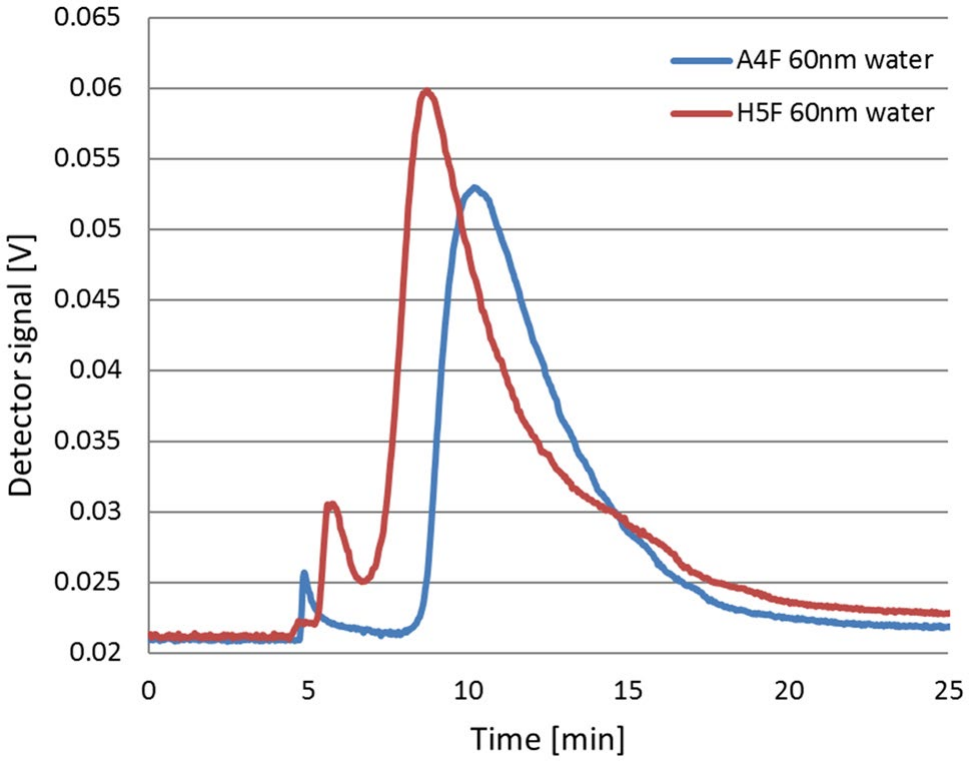
Comparison between AF4 and HF5 analyses for 60 nm particles

By using HF5 technique the recovery rates only for 60 nm particles in two carrier fluids have been reported and accounted to 85.4 ± 0.2 and 77.6 ± 0.3% for water and 1 mM NH_4_NO_3_, respectively. The separation force generated by the cartridge and large particle attraction by membrane made impossible to make meaningful measurements. Lower cross flow rates or other membrane materials should be chosen to achieve separation. HF5 have to be still considered as a alternative for a well-established AF4 fractionation. However, in case of HF5, we have obtained better resolution and intensified peaks. This difference is showed in Fig. 3.

The interaction between the analyte and the membrane is proportional to the ionic strength of the carrier liquid. These effects are greater in the HF5 technique because the cartridge structure is supposed to generate a higher separation force.

### Separation

**Fig. 4:**
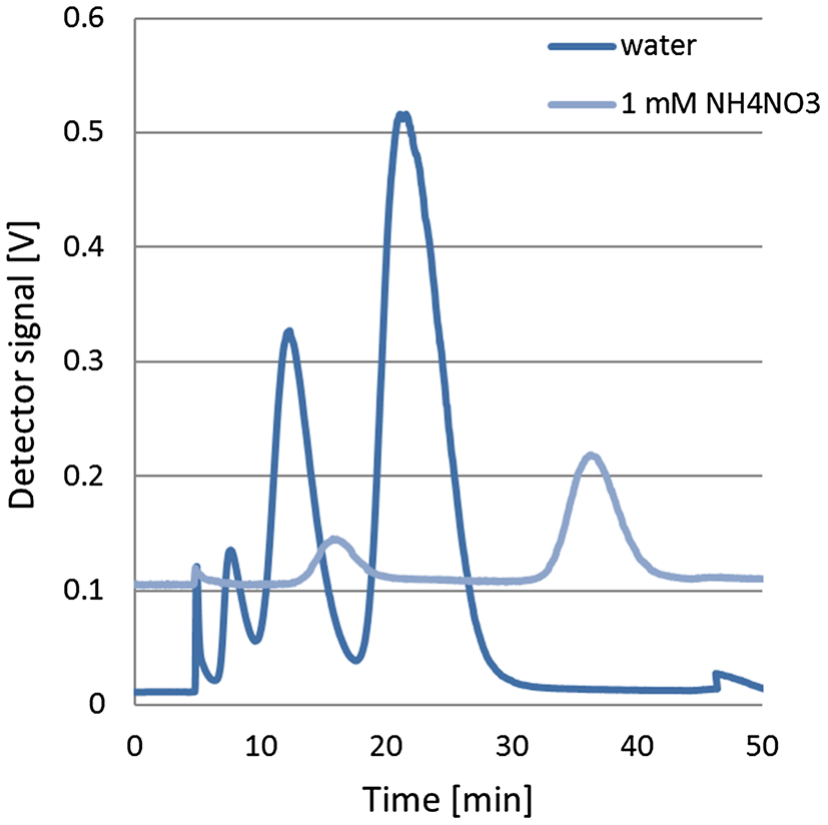
Separation of latex mixture in water and 1 mM NH_4_NO_3_

The latex mixture was separated using the parameters listed in Table 1. Particle separation was carried out using all tested carrier liquids. Under prescribed conditions, the apparatus was unable to carry out separation of the particles suspended in a 10 mM solution. The remaining separation attempts were positive, the results are shown in Fig. 4.

Using water as a carrier liquid, three peaks were obtained which, after evaluation, corresponded to the individual components of the mixture. The separation process took about 40 min. Separation resolution was acceptable. Using 1 mM NH_4_NO_3_ as the carrier liquid, two peaks were obtained corresponding to 60 and 121.9 nm sized particles, respectively. Despite attempts to extend the separation time to 220 min, no peak has been achieved for particles of 356 nm in diameter. This can be caused by, interacting particles with the membrane or agglomeration. Resolution in 1 mM solution compared to water improved at the expense of increased separation time and loss of fraction corresponding to 350 nm.

Schachermeyer et al. [28] also investigated the influence of the ionic strength of the carrier liquid. Separation was obtained for the analysed latex mixture for all carrier fluids used. However, the authors used variable instrument parameters for particular carrier liquids. Constant parameters were used for this study. To achieve full separation, a process should be performed with the change of parameters for the individual carrier fluids. On the contrary, Maisterjahn et al. [29] provided systematic study about the influence of membrane type, composition of carrier liquid and cross flow rate on recovery and retention of silver and gold NPs. They reported great variability of both retention times and recoveries depending on NPs’ type and running conditions, pointing that optimisation of flow-FFF separation is challenging especially for unknown complex samples.

## Conclusions

AF4 and HF5 are powerful tools for sample separation and characterization of nanoparticles in aqueous solution. Direct measurements made with DLS detector indicate a decrease in the hydrodynamic diameter of the nanoparticles along with the increase of ionic strength of the carrier liquid.

The obtained data indicate that the AF4 technique is much more resistant to changes that increase the ionic strength of the carrier liquid. Choosing carrier liquid with the adequate ionic strength is undoubtedly of great importance when performing analysis in both AF4 and HF5. It has to be pointed out that founding optimum composition can be challenging even for the same NPs but with different size.
